# YAP/TAZ links mechanosensing to aging

**DOI:** 10.1093/lifemedi/lnac039

**Published:** 2022-09-12

**Authors:** Xiaolei Cao, Wenqi Wang, Bin Zhao

**Affiliations:** The MOE Key Laboratory of Biosystems Homeostasis & Protection, Zhejiang Provincial Key Laboratory for Cancer Molecular Cell Biology, and Innovation Center for Cell Signaling Network, Life Sciences Institute, Zhejiang University, Hangzhou 310058, China; Center for Life Sciences, Shaoxing Institute, Zhejiang University, Shaoxing 321000, China; Cancer Center, Zhejiang University, Hangzhou, Zhejiang 310058, China; Department of Developmental and Cell Biology, University of California, Irvine, CA 92697, USA; The MOE Key Laboratory of Biosystems Homeostasis & Protection, Zhejiang Provincial Key Laboratory for Cancer Molecular Cell Biology, and Innovation Center for Cell Signaling Network, Life Sciences Institute, Zhejiang University, Hangzhou 310058, China; Center for Life Sciences, Shaoxing Institute, Zhejiang University, Shaoxing 321000, China; Cancer Center, Zhejiang University, Hangzhou, Zhejiang 310058, China

Aging is a progressive functional decline of an organism throughout life [1]. During aging, molecular, and cellular damages accumulate over time, leading to loss of physical and mental capacity, and increased vulnerability to diseases and death. Although a wealth of studies have proposed several hallmarks of aging, such as cellular senescence and mitochondrial dysfunction [1], a major challenge in the field is to understand how these aging-related alterations are initiated and coordinated to contribute to aging *in vivo*. Answering this key question will help to develop approaches for treating aging-related degeneration to improve human health.

Cellular senescence is defined as a stable state of cell cycle arrest induced by cellular stress, accompanied with inflammatory, secretory phenotypes [2]. Recently, cellular senescence has emerged as a fundamental mechanism of aging [2]. By comparing the ratio of senescent cells between young and aged mice using γ-H2A.X foci and senescence-associated β-galactosidase activity as markers, enriched senescent cells were detected in aged liver, skin, lung, and spleen, but not in heart, skeletal muscle, and kidney [3], suggesting tissue-specific mechanisms of aging. Whether cellular senescence occurs in a sequential order in tissues remains unclear. Nevertheless, selective clearance of senescent cells using senolytic approaches alleviated physical tissue dysfunctions in clinical trials [2].

During aging, extracellular matrix (ECM) integrity is decreased due to collagen fragmentation, glycation, crosslinking, and protein aggregation, leading to changes of physical, mechanical, and architectural properties of the ECM [4]. Furthermore, regulation of cellular senescence by mechanical stress was widely reported. However, our understanding of how cells sense and respond to mechanosignals is still limited, despite the discoveries of key sensors such as stretch-sensitive channels from the Piezo family [5] and effectors such as the Yes-associated protein (YAP) and its paralog transcriptional co-activator with PDZ-binding motif (TAZ, also known as WWTR1) [6]. YAP/TAZ are major effectors of the Hippo pathway, an evolutionarily conserved pathway originally described to play critical roles in limiting organ size. The Hippo pathway is comprised with a kinase cascade, where MST1/2 kinases phosphorylate and activate LATS1/2 kinases, which directly phosphorylate YAP/TAZ on multiple sites resulting in their cytoplasmic retention and degradation. When the Hippo pathway is inactive, YAP/TAZ binds to transcription factors like TEAD family proteins to induce the transcription of target genes, including regulators of cell cycle, apoptosis, and cell differentiation. Interestingly, YAP/TAZ was found to robustly respond to mechanosignals [6]. Generally, conditions of high mechanical force, including stiff ECM and high cytoskeletal tension, activate YAP/TAZ; whereas low mechanical force inhibits them. Although it has been initially reported that YAP regulation by mechanical signals is independent of LATS1/2, later studies suggest that LATS1/2 is involved in YAP/TAZ regulation by the actin cytoskeleton [7]. The mechanosensing properties of the Hippo pathway and YAP/TAZ distinguished them from other developmental pathways.

In this report, the Piccolo group found that YAP/TAZ activities were declined during physiological aging primarily in stromal cells, exemplified by dermal fibroblasts and aortic vascular smooth muscle cells (vSMCs), but not in epithelial cells, neurons, or lymphocytes. This decline was associated with a progressive decrease of actomyosin tension. The authors observed a reduced nuclear localization of YAP/TAZ but unchanged YAP phosphorylation, thus proposed an irrelevant role of the canonical Hippo pathway in this process. However, expression of YAP-S127A (a Hippo pathway phosphorylation-deficient mutant of YAP) in skin fibroblasts or vSMCs successfully antagonized the aging phenotypes. Notably, if the aging-induced decline of nuclear YAP/TAZ was through a mechanism independent of the canonical Hippo pathway, it should still be able to suppress YAP-S127A. Although the premature aging phenotypes induced by *YAP/TAZ* knockout were striking, further genetic evidence is needed to clarify the involvement of the canonical Hippo pathway in aging of stromal cells.

**Figure 1.**
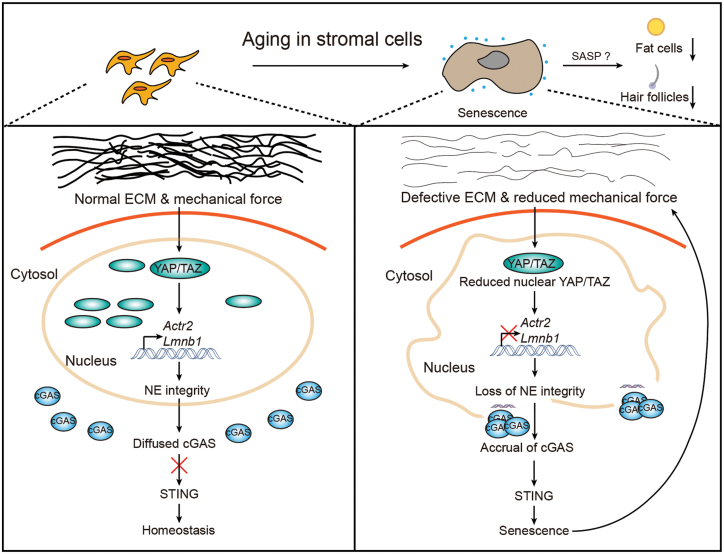
Illustration of the roles of YAP/TAZ in preventing aging by preserving NE integrity in stromal cells. During aging, loss of nuclear YAP/TAZ due to reduced tissue mechanical force results in loss of NE integrity, which is followed by exposure of DNA to cytoplasm and cGAS-STING activation, leading to cellular senescence, and aging phenotypes.

How does YAP/TAZ prevent premature aging? An unexpected finding of this study was the regulation of nuclear envelope (NE) integrity by YAP/TAZ. It was reported that the NE plays a central role in mechanosensing [1], impinging on the expression of Lamin A as a “mechanostat.” Lamin A/C are intermediate filaments localized on the nucleoplasmic surface of the NE. Lamin A has been implicated in physiological aging, as its mutation is known to cause Progeria (or Hutchinson-Gilford syndrome) [1], a severe syndrome of early aging. Thus, the NE was intimately linked to both mechanosensing and aging. Through transcriptional profiling and a functional screen, this report identified two new YAP/TAZ target genes: *Lamin B1* (*Lmnb1)*, which encodes a structural component of the nuclear lamina, and *actin related protein 2* (*Actr2*), which encodes a core component of the Arp2/3 actin nucleation complex. Further experiments proved that proper levels of Arp2 are required for the assembly of perinuclear actin cap, which maintains nuclear shape by forming a dome-like structure wrapping around the apical surface of the nucleus, while the contribution of *Lmnb1* downstream of YAP/TAZ to NE integrity was relatively minor. During aging, loss of nuclear YAP/TAZ results in reduced expression of *Actr2* and *Lmnb1*, which impairs NE integrity to expose DNA to the cytoplasm (Fig. 1).

Cyclic GMP-AMP synthase (cGAS) is recognized as the main sensor for cytoplasmic double-stranded DNA in mammalian cells [8]. Once activated, cGAS produces a second messenger cGAMP, which in turn activates the stimulator of interferon genes (STING) and its downstream transcriptional effects. The cGAS-STING pathway was mostly studied in the context of innate immune response, but it was also identified as a leading inducer of senescence-associated secretory phenotype (SASP) both *in vitro* and *in vivo* [8]. In line with this notion, the authors found that the cGAS-STING pathway-mediated SASP was induced upon the loss of NE integrity and YAP/TAZ activities during aging (Fig. 1). Knockout of *YAP/TAZ* triggers cGAS activation in dermis and aortic wall. Moreover, mechanical inhibition of YAP/TAZ led to activation of cGAS both *in vitro* (in cultured cell on soft ECM or small adhesive areas) and *in vivo* (in *Fbn1*-mutant aortic vSMCs). Importantly, the premature aging traits including *YAP/TAZ* ablation-induced SASP in young mice were largely prevented by genetic or pharmaceutical inactivation of STING. These facts indicate that aberrant activation of the cGAS-STING pathway drives senescence and aging-related tissue degeneration upon YAP/TAZ inactivation.

This is an interesting study, as it not only reveals the mechanism by which the cGAS-STING pathway is regulated by YAP/TAZ, but also links mechanosensing to aging. However, many questions remain to be answered. The first one is about the tissue specificity of YAP/TAZ-mediated control of aging. While YAP/TAZ is also expressed in other tissues, including epithelial cells, their activities were not declined following aging. This could be due to different remodeling of ECM in these tissues during aging. Thus, it will be interesting to examine both the mechanism of aging-related senescence in these tissues and the benefits to the organism when differential mechanisms are adopted. Second, it is unclear how these mechanisms cross-talk between stromal and other tissues during aging. It was observed that knockout of *YAP/TAZ* in dermal fibroblasts also led to non-cell-autonomous reduction of subcutaneous fat and reduced density of hair follicles, two well-known traits of aging skin (Fig. 1), but the underlying mechanism was unknown. It should be noted that in contrast to stromal cells, the aged niche of hair follicle stem cells displayed increased mechanical stress due to widespread alterations in ECM composition, which could be recapitulated by increasing basement membrane stiffness *in vitro*. These facts further highlight the complex roles of mechanosignals in aging, which deserves further investigation.

Another question is whether the cGAS-STING pathway could play additional roles downstream of YAP/TAZ in other contexts, e.g. in cardiac fibroblasts during heart regeneration and fibrosis, and in cancer cells with oncogene-induced senescence. Nevertheless, these new findings could be more relevant to the known roles of YAP/TAZ in innate immunity. For example, viral infection activates IKKε, which phosphorylates YAP and promotes its degradation in the lysosome [9]. On the other hand, YAP/TAZ inhibits antiviral response by preventing the K63-linked ubiquitination of TBK1, and directly disrupted its interaction with substrates, such as STING and MAVS [10]. It will be interesting to further investigate whether and how the newly revealed YAP/TAZ-dependent NE integrity can cooperate with these mechanisms in innate immunity, and whether mechanosensing could also play a role in this biological process.

Findings of this report suggest that sustaining YAP/TAZ activity and inhibition of STING are potential anti-aging approaches. While such agents are already under development by various entities, potential side effects should be a primary consideration regarding their application.
